# Delphinidin induced protective autophagy via mTOR pathway suppression and AMPK pathway activation in HER-2 positive breast cancer cells

**DOI:** 10.1186/s12885-018-4231-y

**Published:** 2018-03-27

**Authors:** Jingyao Chen, Yanfeng Zhu, Weiwei Zhang, Xiaoli Peng, Jie Zhou, Fei Li, Bin Han, Xin Liu, Yu Ou, Xiaoping Yu

**Affiliations:** Department of Public Health, Chengdu Medical College, 783 Xindu Avenue, Xindu, Chengdu, Sichuan 610500 People’s Republic of China

**Keywords:** Delphinidin, Apoptosis, Autophagy, Breast cancer

## Abstract

**Background:**

We have previously demonstrated the anticancer effect of anthocyanins. In this study, we explored the biological activities of delphinidin, the most common of the anthocyanidin monomers, that were related to autophagy in HER-2 positive breast cancer MDA-MB-453 and BT474 cells.

**Methods:**

The effects of various doses of delphinidin on the proliferation and apoptosis of MDA-MB-453 and BT474 cells were analysed. Autophagy was identified as a critical factor that influenced chemotherapy, and the autophagic mechanism in delphinidin-treated cells was investigated. The autophagy inhibitors, 3-MA and BA1, were used to analyse the effects of autophagy inhibition.

**Results:**

Delphinidin inhibited proliferation, promoted apoptosis, and induced autophagy in MDA-MB-453 and BT474 cells in a dose-dependent manner. The inhibition of autophagy enhanced the delphinidin-induced apoptosis and antiproliferative effect in both HER-2 positive breast cancer cells. In addition, delphinidin induced autophagy via suppression of the mTOR signalling pathway and activation of the AMPK signalling pathway in HER-2 positive breast cancer cells.

**Conclusions:**

Collectively, the results showed that delphinidin induced apoptosis and autophagy in HER-2 positive breast cancer cells and that autophagy was induced via the mTOR and AMPK signalling pathways. The suppression of autophagy promoted the anticancer effects of delphinidin.

## Background

Breast cancer, the most common cancer in Chinese women and the second most common female cancer worldwide [1], presents a serious threat to women’s health. The incidence of human epidermal growth factor receptor-2 (HER-2)-positive breast cancer comprises 23% of all breast cancer types and results in a poor prognosis. However, the development of an effective therapy for HER-2-positive breast cancer has proceeded slowly; no widely recognized treatment programmes have yet been accepted [2]. The side effects of traditional therapeutic strategies have driven the development of natural compounds as new therapeutic drugs and promoted the urgent exploration of other anticancer strategies.

Delphinidin, an anthocyanidin monomer, exerts the strongest antioxidative efficiency of all anthocyanidins in the human diet [3]. Our previous studies confirmed the anticancer activities of anthocyanidin and delphinidin-3-glucoside against breast cancer [4–6]. However, although delphinidin is a major bioactive component of anthocyanidin, the mechanisms responsible for its effects against breast cancer remain poorly defined. Therefore, the present study aimed to provide further exploration of the anticancer effects of delphinidin.

Autophagy is shown to act in parallel with apoptosis and serves as an alternative mechanism to cell death when apoptosis is inhibited [7]. However, a number of studies have suggested that autophagy induces protective effects against malignancy in the presence of cellular stress [8, 9]. Delphinidin causes metabolic stress in breast cancer cells and acts a potential anticancer agent. Therefore, the exact role of autophagy induced by delphinidin needs further investigation. Many studies have indicated that autophagy can be activated by the monophosphate-activated protein kinase (AMPK) signalling pathway, which is associated closely with energy metabolism [10, 11]; additionally, it is known that the classic AKT/protein kinase B-mammalian target of rapamycin (mTOR) signalling pathway initiates the vesicular double-membrane formation process of autophagy [12]. Moreover, Atg5, a critical protein that mediates the expansion of autophagosomes, can be cleaved in response to metabolic stress and may even be the switch from autophagy to apoptosis [13].

This study reported novel functions of delphinidin: the induction of apoptosis and protective autophagy in HER-2-positive MDA-MB-453 and BT474 breast cancer cells and the induction of autophagy through the modulation of the mTOR and AMPK signalling pathways. Our findings suggest potential clinical applications in which autophagic inhibition sensitizes cells to the anticancer effect of delphinidin. This research may support the future development of delphinidin or other phytochemicals, as a novel anticancer drug.

## Methods

### Cell culture and treatment

HER-2-positive breast cancer cells (MDA-MB-453 and BT474) were purchased from the Chinese Academy of Sciences (Shanghai, China). MDA-MB-453 cells were cultured in L-15 medium (Gibco, USA, 11415–064) supplemented with 10% foetal bovine serum (Millipore, USA, ES-009-B) at 37 °C in a humidified atmosphere containing 100% O_2_. BT474 cells were cultured in 1640 medium (Gibco, USA, A1049101) supplemented with 10% foetal bovine serum (Millipore, USA, ES-009-B), at 37 °C in a humidified atmosphere with 5% CO_2_. Delphinidin was dissolved in dimethyl sulphoxide (DMSO). The final concentration of DMSO (0.1% volume) was added to medium as a control. Where used, 3-methyladenine (3-MA) (5 mM) was dissolved in phosphate-buffered saline (PBS) and pretreated for 2.5 h before delphinidin administration. Bafilomycin A1 (BA1) (160 nM) was dissolved in DMSO and added 8 h before sample collection and observation.

### Antibodies and reagents

Alexa Fluor 488 Phalloidin (8878), caspase-3 (9665 s), caspase-9 (9502), light chain 3 (LC3)-I/II (2775), AKT (4685), phospho-AKT (4051), mTOR (2972), phospho-mTOR (5536), eukaryotic initiation factor 4E (eIF4E, 2067), phospho-eukaryotic initiation factor 4E (p-eIF4E, 9741), ribosomal protein S6 kinase (p70s6k) (9202), phospho-ribosomal protein S6 kinase, (p-p70s6k, 9206), serine/threonine kinase LKB1/STK11 (3047), phospho-LKB1 (3482), forkhead box O3a (FOXO3a, 2497), AMPK (2532), phospho-forkhead box O 3a (p-FOXO3a, 9465), UNC-51-like kinase-1 (ULK1, 4776), phospho-UNC-51-like kinase-1 (ULK1, 12,753), anti-rabbit secondary antibody (7054), and anti-mouse secondary antibody (7056) were purchased from Cell Signaling Technology, USA. Atg5 (ab78073) and phospho-AMPK (ab195946) were purchased from Abcam, UK. β-Actin (TA-09) was purchased from ZSGB-BIO, China. Other reagents were obtained from the following sources as required: delphinidin (Sigma, USA, 43725); 3-MA (Sigma, USA, M9281); BA1 (Cayman, USA, 88899–55-2); DMSO (Sigma, USA, D2650); and cell counting kit-8 (CCK-8) (Dojindo, Japan, CK04–20).

### Cell proliferation assay

MDA-MB-453 and BT474 cells were seeded into 96-well culture plates for 24 h and then treated with delphinidin for 48 h. CCK-8 assay was used to evaluate the viability of cells. The absorbance of the solutions in the wells was read at 450 nm in a microplate reader (BioTek, China, Powerwave XS).

### Terminal deoxynucleotidyl transferase dUTP nick-end labelling assay

Terminal deoxynucleotidyl transferase dUTP nick-end labelling (TUNEL) staining was performed using the DeadEnd Fluorometric TUNEL system in accordance with the manufacturer’s instructions (Promega, USA, G3250). The samples were observed and recorded by using a fluorescence microscope (Olympus, Japan, IX71). The nuclei stained with bright green fluorescence were considered to be TUNEL-positive cells.

### Immunoblotting

The adherent cells were collected in radio immunoprecipitation assay (RIPA) buffer by using a cell scraper. A protein assay kit (Thermo, USA, 23227) was used to quantify protein concentration. The samples were separated by sodium dodecyl sulphate-polyacrylamide gel electrophoresis and the proteins were transferred to polyvinylidene fluoride membranes. Non-fat milk solution (5%) was used to block the membranes. After blocking, the membranes were treated with the primary antibodies at 4 °C overnight, washed three times in TBST, and then incubated with the secondary antibodies for 2 h at 37 °C. Finally, chemiluminescence reagents (Millipore, WBKLS0500) were used to visualize the stained proteins.

### Transmission electron microscopy

The samples were fixed for 2 h with 2.5% glutaraldehyde in phosphate buffer, washed three times in 0.1 M phosphate buffer, fixed with 1% osmic acid for 2 h, and washed again. The samples were dehydrated with graded alcohol (50, 70, and 90%), dehydrated with 90% alcohol and 90% acetone at 4 °C, and finally dehydrated with 100% acetone at 18–21 °C (room temperature). The samples were embedded in a mixture of acetone and epoxy resin. The samples were solidified at different temperatures (37, 45, and 60 °C), sliced on an ultramicrotome (Leica, Germany, UC7), double stained with 3% uranyl acetate and lead citrate, and examined under a transmission electron microscope (JEOL, Japan, JEM-1011).

### Immunofluorescence analysis

The cells were grown on coverslips in 6-well plates, administered delphinidin at different concentrations for 48 h, fixed in 4% formaldehyde, washed, and permeabilised with 0.1% Triton X-100. The samples were washed again and sequentially incubated with anti-LC3 primary antibody and Alexa Fluor 488 Phalloidin secondary antibody. The coverslips were then infiltrated with 60% glycerol and immediately examined under a fluorescence microscope (Olympus, IX71).

### GFP-LC3 transient transfection

The cells were seeded on coverslips in 6-well plates for 24 h and then transfected with the apEX-GFP-hLC3WT plasmid (Addgene, USA, 24987) by using Lipofectamine 2000 (Invitrogen, USA, 11668019) based on the manufacturer’s protocol. After 24 h, the transfected cells were treated with different concentrations of delphinidin for 48 h and washed three times with PBS. The washed samples were fixed with 4% paraformaldehyde for 15 min and then washed again. A solution of 50% glycerol was used to infiltrate the coverslips and observe the distribution of green fluorescent protein (GFP)-LC3 punctate dots in cells. Finally, the images were photographed by using a fluorescence microscope (Olympus, Japan, IX71). The GFP-LC3 punctate dot assay was repeated three times.

### Statistical analyses

All experimental data were presented as the mean ± standard error of the mean and each experiment was performed at least three times. The statistical analyses were performed by one-way ANOVA using SPSS version 21.0 (SPSS, IL, USA). Values of *P* < 0.05 were considered statistically significant.

## Results

### Delphinidin inhibited proliferation and promoted apoptosis in HER-2 positive MDA-MB-453 and BT-474 breast cancer cells

**Fig. 1:**
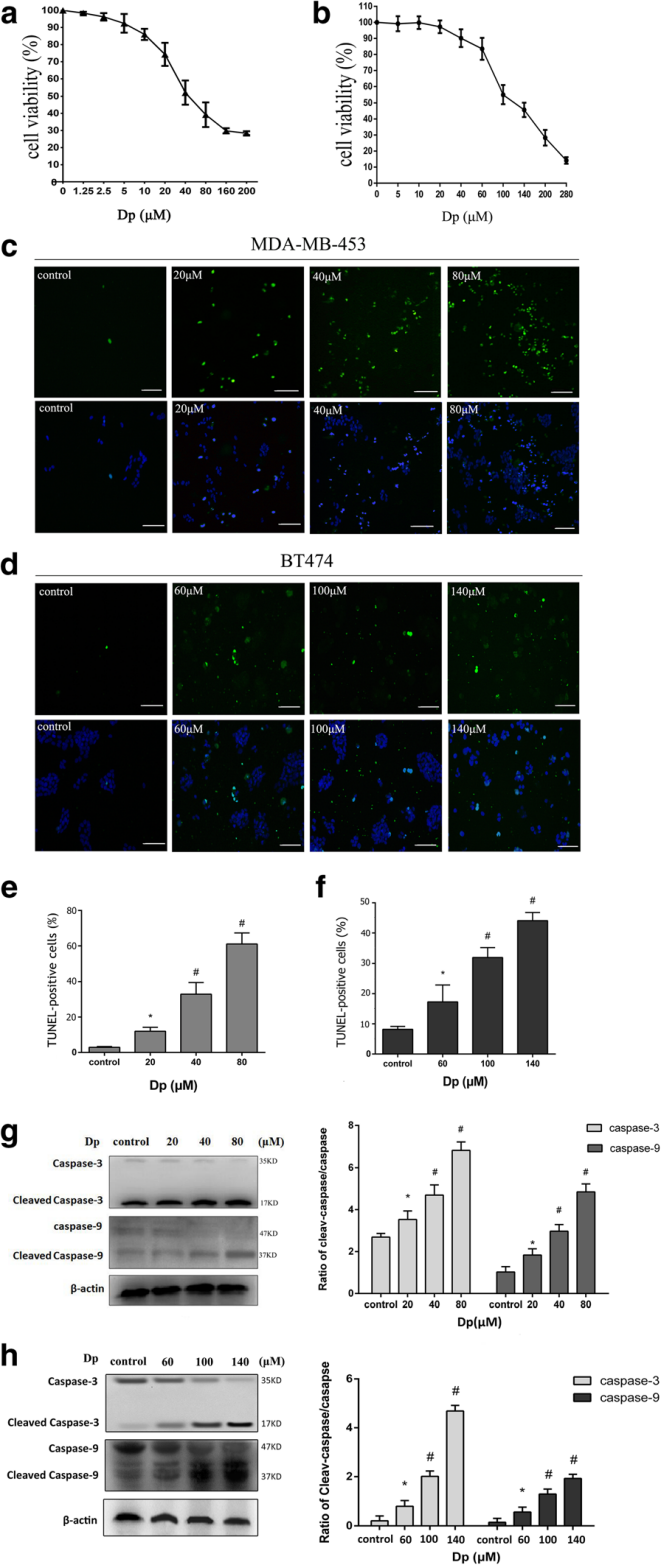
Delphinidin induced apoptosis in MDA-MB-453 and BT474 cells. **a**: CCK-8 assay to evaluate MDA-MB-453 cell viability; (**b**): CCK-8 assay to evaluate BT474 cell viability; (**c**): Expression of TUNEL-positive MDA-MB-453 cells (× 200). **d** Expression of TUNEL-positive BT474 cells (× 200). **e** Statistical graph of TUNEL-positive MDA-MB-453 cells. **f** Statistical graph of TUNEL-positive BT474 cells. **g** Expression of protein cleaved caspase-3 and cleaved caspase-9 in MDA-MB-453 cells. Statistical analysis of the cleaved caspase/caspase ratio. **h** Expression of protein cleaved caspase-3 and cleaved caspase-9 in BT474 cells. Statistical analysis of the cleaved caspase/caspase ratio. Dp: Delphinidin; control: DMSO (0.1%). *P < 0.05 vs control group; ^#^*P* < 0.05 vs Dp group

The effect of delphinidin on HER-2-positive breast cancer cells was explored by the determination of cell viability using the CCK-8 assay. As shown in Fig. 1a and b, the treatment with delphinidin inhibited the proliferation of MDA-MB-453 and BT474 cells in a dose-dependent manner. The IC50 values of delphinidin against MDA-MB-453 and BT474 cells were approximately 40 μM and 100 μM, respectively. In order to explore the mechanism underlying the inhibition of proliferation, the apoptotic effect of delphinidin in HER-2 positive breast cancer cells was examined by using the TUNEL assay. The DNA fragmentation of the experimental groups was clearly increased in comparison with that of the control group in a dose-dependent manner (Fig. 1c-f). To explore whether a caspase-dependent phenomenon was involved in delphinidin-induced apoptotic cell death, activation of caspase-3 and caspase-9 was investigated. Cleaved caspase-9 and cleaved caspase-3 were both upregulated by delphinidin treatment, but caspase-9 and caspase-3 were gradually reduced (Fig. 1g and h). This implied that delphinidin induced caspase-3- and caspase-9-dependent apoptosis in HER-2 positive breast cancer cells. Collectively, the data suggested that delphinidin induced apoptosis in a dose-dependent manner in HER-2 positive breast cancer cells.

### Delphinidin induced autophagy in HER-2 positive breast cancer cells

**Fig. 2:**
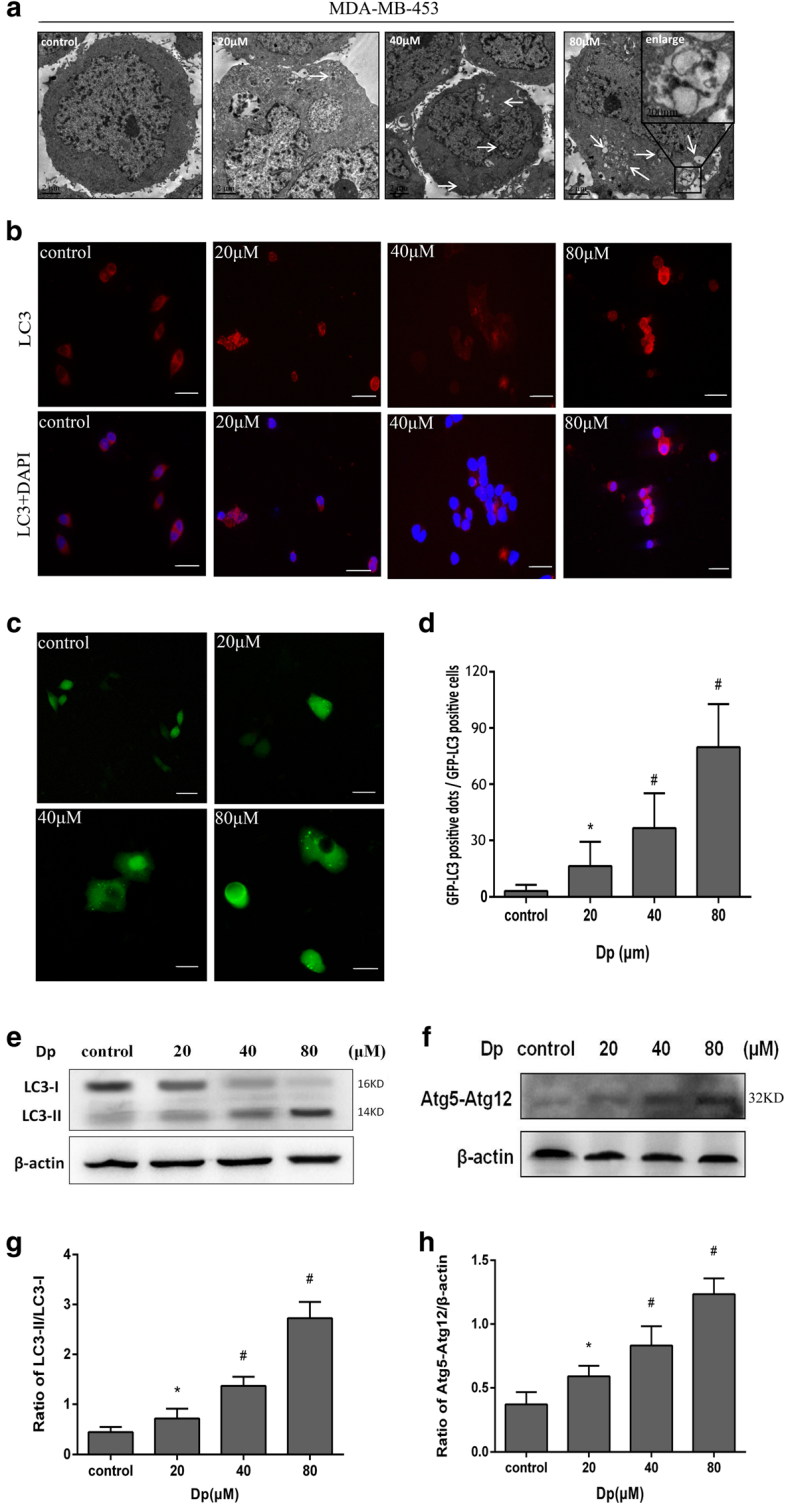
Delphinidin induced autophagy in MDA-MB-453 cells. **a** Representative transmission electron microscopic images of MDA-MB-453 cells treated with DMSO (0.1%) or delphinidin (20, 40, and 80 μM) for 48 h. Arrows: autophagic vacuoles. **b** LC3 immunofluorescence-positive dots in MDA-MB-453 cells (× 400). **c** Expression of GFP-LC3-positive punctate dot (× 400). **d** Statistical graph of GFP-LC3-positive dots. **e** Expression of the LC3-II protein. **f** Expression of the Atg5-Atg12 conjugated protein; (**g**): Statistical analysis of the LC3-II/LC3-I ratio. **h** Statistical analysis on the ratio of Atg5-Atg12/β-actin. Dp: Delphinidin. *P < 0.05 vs control group; ^#^*P* < 0.05 vs Dp group

**Fig. 3:**
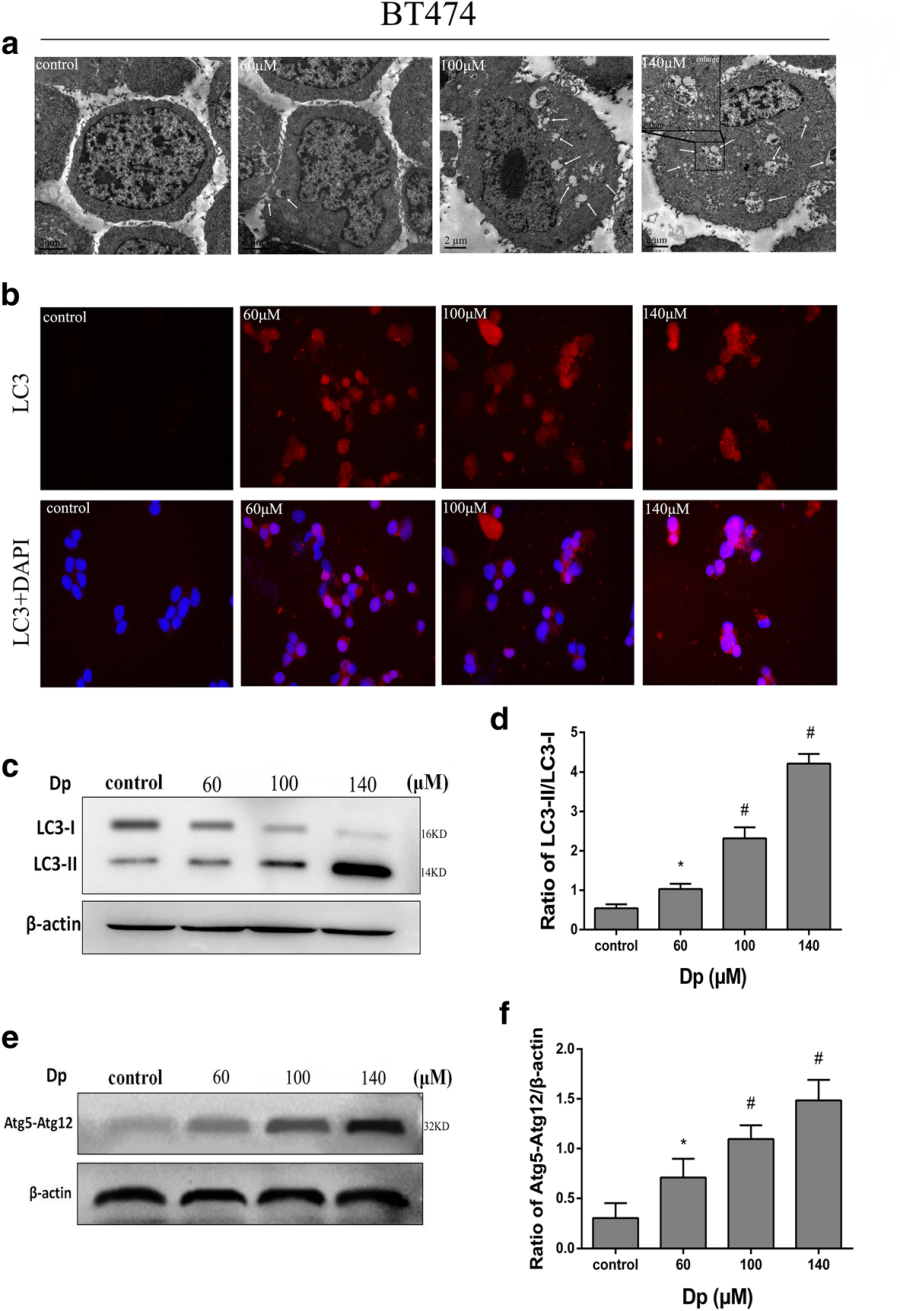
Delphinidin induced autophagy in BT474 cells. **a** Representative transmission electron microscopic images of BT474 cells treated with DMSO (0.1%) or delphinidin (60, 100, and 140 μM) for 48 h. Arrows: autophagic vacuoles. **b** LC3 immunofluorescence-positive dots in BT474 cells (× 400). **c** Expression of LC3-II protein. **d** Statistical analysis of the LC3-II/LC3-I ratio. **e** Expression of the Atg5-Atg12 conjugated protein; (**f**): Statistical analysis on the ratio of Atg5-Atg12/β-actin. Dp: Delphinidin. *P < 0.05 vs control group; ^#^*P* < 0.05 vs Dp group

To elucidate the anticancer effect of delphinidin, we explored the induction of autophagy by delphinidin in both HER-2 positive breast cancer cell lines. The formation of autophagosomes was considered to be the golden standard of autophagic identification [14]. Therefore, we evaluated autophagic activity by using transmission electron microscopy. Autophagic vacuoles were observed in delphinidin treated MDA-MB-453 (20, 40, and 80 μM) and BT474 (60, 100, and 140 μM) cells compared with the control group (Figs. 2a and 3a). In particular, at 80 μM MDA-MB-453 and 140 μM BT474, we observed that various characteristic membranes of autophagosomes appeared in the cytoplasm of the delphinidin-treated groups.

The protein LC3-II is recognized as an autophagosomal marker that locates autophagosomes [15]. Upon the induction of autophagy, the lipidated LC3 is transformed from LC3-I into LC3-II. A combination of assays (immunofluorescence assay, formation of punctate dots with GFP-MAPLC3B, and immunoblot) was used to detect transformed LC3. The results of the immunofluorescence assay (shown in Figs. 2b and 3b) showed that delphinidin induced condensed bright red fluorescent dots in both cells. The GFP-LC3 transient transfection assay is a classical experiment to detect autophagosomes. MDA-MB-453 cells were transfected transiently with the pEGFP-LC3 plasmid. One green LC3 punctate dot was representative of an autophagosome. It was shown in Fig. 2c that delphinidin increased the expression of green punctate dots in the cytoplasm, which signified that delphinidin enhanced the induction of autophagy in MDA-MB-453 cells. The average number of GFP-LC3 punctate dots in the cells gradually increased with an increase in delphinidin concentration (Fig. 2d). Moreover, immunoblot analysis was performed to detect whether delphinidin administration induced the transformation of full-length LC3-I into LC3-II. The aggregation of LC3-II in the cells is shown in Fig. 2e and g; the transformed LC3-II was associated with an increase in delphinidin concentration in MDA-MB-453 cells. The same phenomenon and trend were seen in BT474 cells (Fig. 3c and d).

A series of autophagy genes was involved in autophagosome mediation during the induction of autophagy. Atg5, a key autophagic regulatory protein, is essential for the formation of autophagic vacuoles. The Atg5-Atg12 conjugate complex disassociates upon the formation of autophagosomes [16]. To examine whether the complex was activated and displayed similar properties to delphinidin-induced autophagy, immunoblot analyses were performed to measure the expression of Atg5-Atg12 in MDA-MB-453 cells. In the cells, an increase in delphinidin concentration was found to upregulate the expression of the conjugate complex protein (Fig. 2f and h). The same phenomenon and trend were seen in BT474 cells (Fig. 3e and f).

### Autophagic inhibition enhanced delphinidin-induced suppression of proliferation in HER-2 positive breast cancer cells

**Fig. 4:**
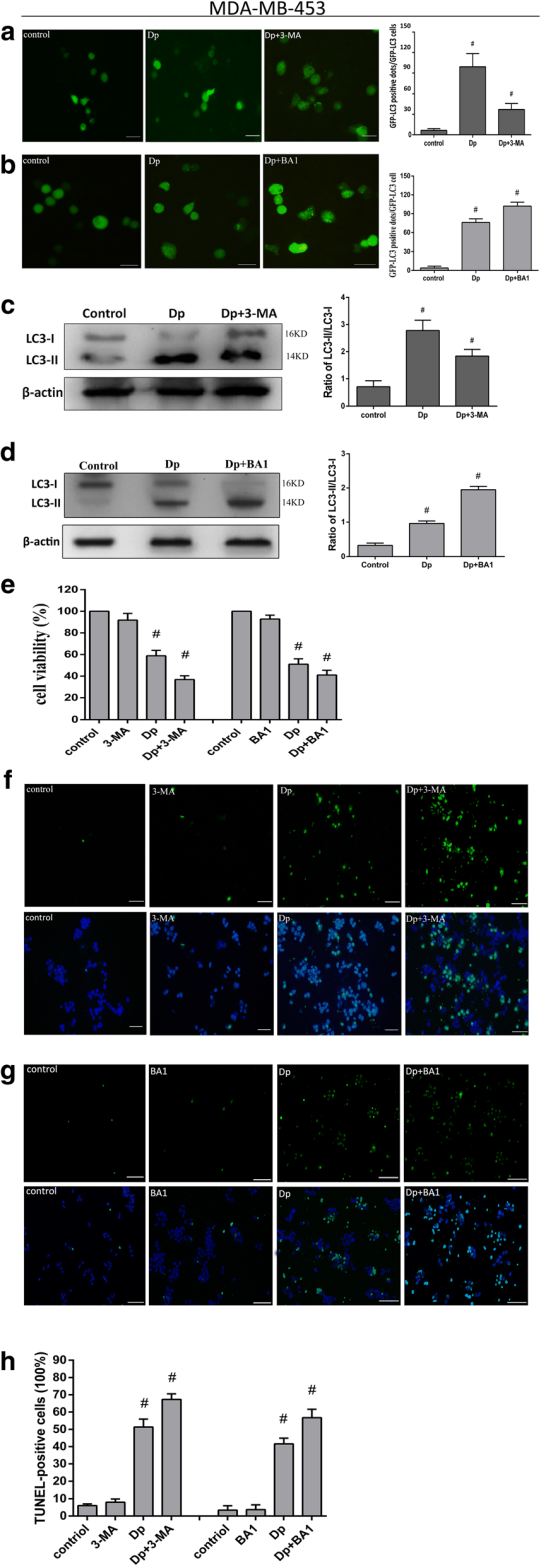
Autophagic inhibition promoted delphinidin-induced apoptosis in MDA-MB-453 cells. **a** Expression of GFP-LC3-positive punctuate dots under autophagy inhibition by 3-MA (× 400). Statistical graph of GFP-LC3-positive dots. **b** Expression of GFP-LC3-positive punctuate dots under autophagy inhibition by BA1 (× 400). Statistical graph of GFP-LC3-positive dots. **c** The expression of LC3-II protein under autophagy inhibition by 3-MA. Statistical analysis of the LC3-II/LC3-I ratio. **d** The expression of LC3-II protein under autophagy inhibition by BA1. Statistical analysis of the LC3-II/LC3-I ratio. **e** CCK-8 assay to evaluate MDA-MB-453 cells viability under autophagy inhibition by 3-MA and BA1 respectively. **f** Expression of TUNEL-positive MDA-MB-453 cells after autophagic inhibition by 3-MA. **g** Expression of TUNEL-positive MDA-MB-453 cells after autophagic inhibition by BA1. **h** Statistical graph of TUNEL-positive MDA-MB-453 cells under autophagy inhibition by 3-MA and BA1. The concentration of Dp was 80 μM. Control: DMSO (0.1%). 3-MA: 5 mM. BA1: 160 nM. Dp: delphinidin. Data were obtained from three independent experiments. *P < 0.05 vs control group

**Fig. 5:**
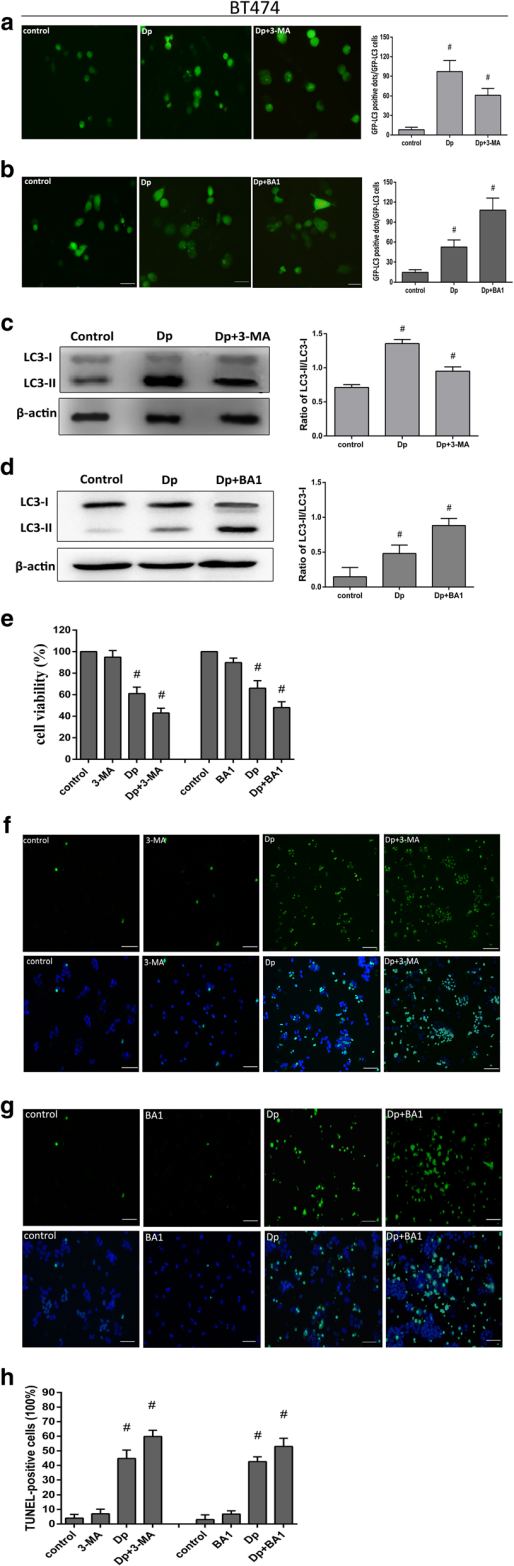
Autophagic inhibition promoted delphinidin-induced apoptosis in BT474 cells. **a** Expression of GFP-LC3-positive punctuate dots under autophagy inhibition by 3-MA (× 400). Statistical graph of GFP-LC3-positive dots. **b** Expression of GFP-LC3-positive punctuate dots under autophagy inhibition by BA1 (× 400). Statistical graph of GFP-LC3-positive dots. **c** The expression of LC3-II protein under autophagy inhibition by 3-MA. Statistical analysis of the LC3-II/LC3-I ratio. **d** The expression of LC3-II protein under autophagy inhibition by BA1. Statistical analysis of the LC3-II/LC3-I ratio. **e** CCK-8 assay to evaluate BT474 cells viability under autophagy inhibition by 3-MA and BA1 respectively. **f** Expression of TUNEL-positive BT474 cells after autophagic inhibition by 3-MA. **g** Expression of TUNEL-positive B474 cells after autophagic inhibition by BA1. **h** Statistical graph of TUNEL-positive BT474 cells under autophagy inhibition by 3-MA and BA1. The concentration of Dp was 140 μM. Control: DMSO (0.1%). 3-MA: 5 mM. BA1: 160 nM. Dp: delphinidin. Data were obtained from three independent experiments. *P < 0.05 vs control group

Many studies have suggested a dual function for autophagy; depending on the stimulus conditions, autophagy could either promote autophagy-associated cell death or induce a protective effect to mediate cancer cell survival [17, 18]. In order to determine the biological role of autophagy, we investigated the conditions of cell proliferation and apoptosis mediated by delphinidin in the presence of the autophagy inhibitors 3-MA and BA1. As an early inhibitor of autophagy, 3-MA prevents the completion of autophagy through disruption of the process of autophagosome membrane formation. We treated cells with either 3-MA or delphinidin alone and in combination. Autophagy was noticeably suppressed by 3-MA, as shown by the number of GFP-LC3 punctate dots and the activity of transformed protein LC3-II in MDA-MD-453 (Fig. 4a and c) and BT474 cells (Fig. 5a and c). As indicated by Figs. 4a and 5a, 3-MA decreased the number of GFP-LC3 punctate dots induced by delphinidin. Figures 4c and 5c show that 3-MA suppressed the transformation of LC3. In addition, when the late inhibitor of autophagy, BA1, was administered to both cells, it was found that BA1 increased the number of GFP-LC3 punctate dots and the conversion of LC3 (Figs 4b, d and 5b, d). As expected, the present results indicated that autophagic inhibition enhanced delphinidin-induced proliferation inhibition in both MDA-MB-453 cells and BT474 cells (Figs 4e and 5e). In addition, the TUNEL assay was used to evaluate apoptosis after autophagic inhibition. As the 3-MA and BA1-induced autophagic inhibition in MDA-MB-453 and BT474 cells augmented the amount of DNA fragmentation (Figs. 4f-h and 5f-h), it was suggested that autophagic inhibition promoted delphinidin-induced apoptosis. Collectively, the results suggested that autophagic inhibition could enhance the effect of delphinidin on the inhibition of proliferation and the induction of apoptosis in HER-2 positive breast cancer cell lines.

### Suppression of mTOR signalling pathway induced autophagy in HER-2 positive breast cancer cells

**Fig. 6:**
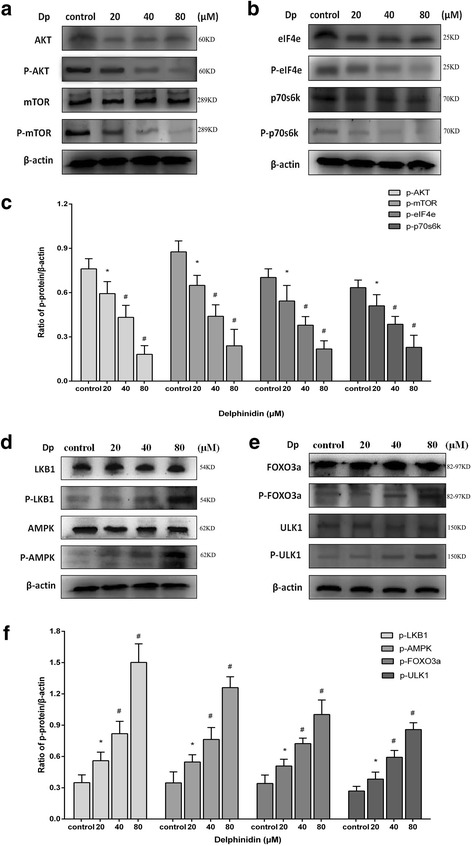
Delphinidin suppressed the mTOR signalling pathway and activated the AMPK signalling pathway in MDA-MB-453 cells. **a** The suppression of upstream proteins from the mTOR signalling pathway, including AKT, phospho-AKT, mTOR, and phospho-mTOR, after the administration of different concentrations of delphinidin for 48 h. **b** The suppression of downstream proteins from the mTOR signalling pathway, including eIF4e, phospho-eIF4e, p70S6K, and phospho-p70S6K, after the administration different concentrations of delphinidin for 48 h. **c** The phosphorylated protein/β-actin ratio in the mTOR signalling pathway. **d** The activity of upstream proteins in the AMPK signalling pathway, including LKB1, phospho-LKB1, AMPK and phospho-AMPK, after administration of different concentrations of delphinidin for 48 h. **e** The activity of downstream proteins from the AMPK signalling pathway including FOXO3a, phospho-FOXO3a, ULK1, and phospho-ULK1 after administration of different concentrations of delphinidin for 48 h. **f** The phosphorylated protein/β-actin ratio in the AMPK signalling pathway. Dp: Delphinidin. Data were obtained from three independent experiments. *P < 0.05 vs control group; ^#^*P* < 0.05 vs Dp group

**Fig. 7:**
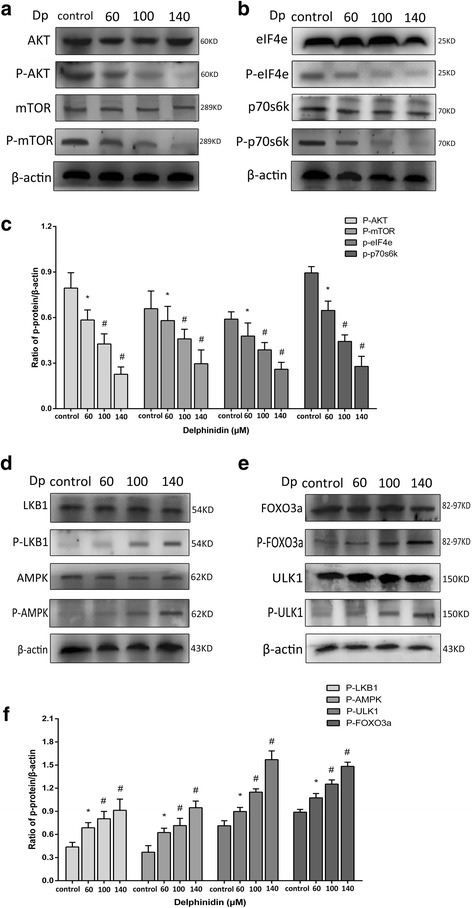
Delphinidin suppressed the mTOR signalling pathway and activated the AMPK signalling pathway in BT474 cells. **a** The suppression of upstream proteins from the mTOR signalling pathway, including AKT, phospho-AKT, mTOR, and phospho-mTOR, after the administration of different concentrations of delphinidin for 48 h. **b** The suppression of downstream proteins from the mTOR signalling pathway, including eIF4e, phospho-eIF4e, p70S6K, and phospho-p70S6K, after the administration different concentrations of delphinidin for 48 h. **c** The phosphorylated protein/β-actin ratio in the mTOR signalling pathway. **d** The activity of upstream proteins in the AMPK signalling pathway, including LKB1, phospho-LKB1, AMPK and phospho-AMPK, after administration of different concentrations of delphinidin for 48 h. **e** The activity of downstream proteins from the AMPK signalling pathway including FOXO3a, phospho-FOXO3a, ULK1, and phospho-ULK1 after administration of different concentrations of delphinidin for 48 h. **f** The phosphorylated protein/β-actin ratio in the AMPK signalling pathway. Dp: Delphinidin. Data were obtained from three independent experiments. *P < 0.05 vs control group; ^#^*P* < 0.05 vs Dp group

The AKT/mTOR signalling pathway has been reported as a negative sensor in autophagy regulation; the phenomenon has been demonstrated in various different cancer cells [19–21]. To explore the mechanism of delphinidin-induced autophagy, we examined the expression of the proteins in mTOR signalling pathway, including AKT, mTOR, eIF4e, and p70s6K, in HER-2 positive breast cancer cells. Delphinidin caused a notable inhibition of phospho-AKT and phospho-mTOR, upstream proteins in the mTOR signalling pathway in MDA-MB-453 and BT474 cells (Figs. 6a and 7a). Moreover, it was shown that the phosphorylation was decreased with an increased dose of delphinidin (Figs. 6c and 7c). It has been reported that downstream proteins of mTOR signalling pathway, p70S6K and eIF4e, were controlled by the mTOR receptor [22]. The objective of the present study was to examine the phosphorylation status of both p70S6K and eIF4e by immunoblot analysis. It was found that delphinidin treatment suppressed the phosphorylation of both p70S6K and eIF4E and that the phosphorylation was decreased by an increase in the dose of delphinidin (Figs. 6b, 7c and 7b, c).

### Activation of AMPK signalling pathway induced autophagy in HER-2 positive breast cancer cells

AMPK, a key energy receptor, regulates cellular energy homeostasis and can be activated by a variety of stimuli, especially by agents that affect the cellular metabolism [23]. After phosphorylation, AMPK is a positive sensor of autophagy from its upstream and downstream proteins. The present study therefore examined whether the AMPK pathway was involved in delphinidin-induced autophagy. Delphinidin treatment resulted in both LKB1 and AMPK phosphorylation in breast cancer cells (Figs. 6d and 7d). Moreover, Figs. 6f and 7f show that the phosphorylation of LKB1 and AMPK was upregulated by an increase in the dose of delphinidin. Downstream proteins of AMPK, including ULK1 and FOXO3a, have been found in addition to the phosphorylation, which play an important role in autophagy and apoptosis [24, 25]. Their activity was also detected in delphinidin-treated breast cancer cells; delphinidin promoted the activation of ULK1 and FOXO3a and this activation was enhanced by an increase in the dose of delphinidin (Figs. 6e, f and 7e, f).

## Discussion

This study on delphinidin and HER-2 positive breast cancer demonstrated that delphinidin promoted antiproliferative effects and apoptosis in human HER-2 positive breast cancer MDA-MB-453 and BT474 cells. Furthermore, it was revealed that delphinidin simultaneously induced autophagy, which promoted cell viability and suppressed apoptotic cell death. The suppression of autophagy using 3-MA and BA1 in HER-2 positive breast cancer cells increased delphinidin-induced apoptosis and antiproliferation. Protective autophagy was induced over a range of treatment doses and was enhanced by an increase in the dose of delphinidin via suppression of the AKT/mTOR/eIF4e/p70s6k signalling pathway and activation of the LKB1/AMPK/ULK1/FOXO3a signalling pathway in HER-2 positive breast cancer cells.

Delphinidin, an anthocyanidin monomer, is prevalent in vegetables and fruits. Our previous research has demonstrated that anthocyanidin exerts an anticancer effect on HER-2 positive MDA-MB-453 breast cancer cells, but displays low cytotoxicity to normal breast cells [4, 5]. As the monomer predominantly responsible for the activity of anthocyanidin, delphinidin has been shown to exert anticancer effects in various cancer models [26, 27]. Consistent with previous reports [26, 27], the present study showed that delphinidin induced antiproliferative effects and apoptosis in human HER-2 positive breast cancer MDA-MB-453 and BT474 cells. These findings are significant for the further development of delphinidin as a clinical chemopreventive agent.

Autophagy exerts contradictory effects in different stages of cancer cell formation. Certain phytochemicals, such as ampelopsin and baicalein, induce protective autophagy that counteract antiproliferative effects and apoptosis [28, 29], whereas others, such as cyclovirobuxine D and *Zanthoxylum* fruit, initiate autophagic cell death that sensitize cells to anticancer effects [30, 31]. The present study demonstrated that delphinidin triggered autophagy and that autophagic inhibition by 3-MA and BA1 markedly enhanced the antiproliferative effects and apoptosis by delphinidin. It was suggested that the autophagy might exert protective efficiency in HER-2 positive breast cancer cells.

Caspase-dependent cell death, a method of apoptosis, is regulated in two main ways: the activation of exogenous death regulators and the endogenous release of cytochrome c. The activation of exogenous death regulators cleaves caspase-3, whereas cytochrome c release upregulates cleaved caspase-9 and cleaved caspase-3 [32, 33]. Consequently, as delphinidin modulated the activation of caspase-3 and -9 in the present study, the cell death was believed to be mediated by the endogenous pathway. Several studies suggested that the endogenous pathway in cancer cells was related to activation of the endoplasmic reticulum stress pathway and the generation of reactive oxygen species [28, 34]. To confirm that delphinidin-induced apoptosis was related to the endogenous pathway, further studies are necessary to detect the activity of the endoplasmic reticulum stress pathway and reactive oxygen species.

Many signalling pathways are involved in the induction of autophagy; however, the mTOR-related pathway, as a pivotal negative sensor of autophagy, is more significant. Many phytochemical compounds regulate autophagy through the mTOR pathway in MCF-7 breast cancer cell models [28, 30]. To reveal the molecular mechanism of delphinidin-induced autophagy, the relationship between delphinidin, autophagy, and the mTOR pathway was explored. It was found that treatment with delphinidin specifically inhibited the AKT branch upstream of mTOR, affecting eIF4e and p70s6k downstream of mTOR phosphorylation, which suggested that delphinidin exerted a negative effect on mTOR activity. The result was similar to the effect of rapamycin, a natural inhibitor of mTOR and agonist of autophagy, on autophagy. It is well known that mTOR is a central pathway in the mediation of cell growth, protein synthesis, survival, and metabolism in response to hormones, nutrients, and other stimuli [35]. The dysfunction of the mTOR pathway in mammary cells often leads to breast carcinogenesis [36]. In the present study, the suppressive effect of delphinidin on HER-2 positive breast cancer cells was shown to occur through the mTOR pathway; thus, the proliferation inhibition and autophagy induced by delphinidin might be attributable to the same pathway.

AMPK, an energy sensor of cells, is associated with the activation of autophagy under the conditions of the catabolic processes of oxidative stress and energy starvation in eukaryotic cells. Previous studies have shown that AMPK activated autophagy through the direct activation of the downstream receptor ULK1, whereas others elucidated that AMPK activated autophagy through the inhibition of mTOR phosphorylation in pancreatic β cells [24, 37]. The present study demonstrated that AMPK activated ULK1, a homolog of yeast ATG1, by phosphorylation at ser317 and a reduction in the activation of mTOR, which indicated the interaction between ULK1 and mTOR in delphinidin-induced autophagy. Shaw proposed that LKB1 and AMPK controlled mTOR signalling and cell growth. Hence, it was thought that the growth inhibition of MDA-MB-453 and BT474 cells induced by delphinidin was related to the activation of LKB1 and AMPK [38]. FOXO3a, a member of forkhead box O (FoxO) family of transcription factors, has been reported to initiate the expression of autophagy-related genes [38]. The present study showed that FOXO3a could be upregulated by the activation of AMPK, resulting in the induction of autophagy, which suggested that FOXO3a probably initiated autophagy-related genes. Several studies have found that the AMPK-FOXO3a axis plays an important role in the regulation of autophagy-related genes in different cell models [39, 40].

## Conclusion

In this study, the cellular responses to delphinidin showed that the induction of autophagy occurred through the antagonization of apoptotic cell death in human HER-2 positive breast cancer MDA-MB-453 and BT474 cells. The mTOR and AMPK signalling pathways were also shown to be involved in delphinidin-induced autophagic induction in MDA-MB-453 and BT474 cells. However, the study did not demonstrate the phenomenon of protective autophagy induced by delphinidin *in vivo*. To validate the rational use of delphinidin in the prevention of various types of cancers, further exploration of the effects of delphinidin in *in vivo* models is necessary.

## Abbreviations

3-MA: 3-methyladenine
AMPK: Monophosphate-activated protein kinase
BA1: Bafilomycin A1
CCK-8: Cell counting kit-8
DMSO: Dimethyl sulphoxide
Dp: Delphinidin
eIF4E: Eukaryotic initiation factor 4E
FOXO3a: Forkhead box O3a
GFP: Green fluorescent protein
LC3: Light chain 3
LKB1/STK11: Serine/threonine kinase
mTOR: Mammalian target of rapamycin
p70s6k: Ribosomal protein S6 kinase
PBS: Phosphate-buffered saline
RIPA: Radio immunoprecipitation assay
TUNEL: Transferase dUTP nick-end labelling
ULK1: UNC-51-like kinase-1
